# Comparative Analysis between the In Vivo Biodistribution and Therapeutic Efficacy of Adipose-Derived Mesenchymal Stromal Cells Administered Intraperitoneally in Experimental Colitis

**DOI:** 10.3390/ijms19071853

**Published:** 2018-06-23

**Authors:** Mercedes Lopez-Santalla, Pablo Mancheño-Corvo, Amelia Escolano, Ramon Menta, Olga Delarosa, Juan M. Redondo, Juan A. Bueren, Wilfried Dalemans, Eleuterio Lombardo, Marina I. Garin

**Affiliations:** 1 Division of Hematopoietic Innovative Therapies, Centro de Investigaciones Energéticas, Medioambientales y Tecnológicas (CIEMAT) and Centro de Investigación Biomédica en Red de Enfermedades Raras (CIBERER-ISCIII), Avda, Complutense, 40, 28040 Madrid, Spain; mercedes.lopezsantalla@ciemat.es (M.L.-S.); juan.bueren@ciemat.es (J.A.B.); 2 Advanced Therapy Unit, Instituto de Investigación Sanitaria Fundación Jiménez Díaz, (IIS-FJD/UAM), Avda, Complutense, 40, 28040 Madrid, Spain; 3 TiGenix SAU, Calle de Marconi, 1, 28760 Tres Cantos, Madrid, Spain; pablo.mancheno@tigenix.com (P.M.-C.); ramon.menta@tigenix.com (R.M.); olga.delarosa@Tigenix.com (O.D.); 4 Gene Regulation in Cardiovascular Remodeling and Inflammation Laboratory, CNIC, Calle de Melchor Fernández Almagro, 3, 28029 Madrid, Spain; amelia.escolano@rockefeller.edu (A.E.); jmredondo@cnic.es (J.M.R.); 5 Laboratory of Molecular Immunology, The Rockefeller University, 1230 York Avenue, NY 10065, USA; 6 TiGenix NV, Romeinse straat 12, 3001 Leuven, Belgium; wilfried.dalemans@tigenix.com

**Keywords:** adipose-derived mesenchymal stem cells, intraperitoneal therapy, biodistribution, efficacy, colitis

## Abstract

Mesenchymal stem cells (MSCs) have emerged as a promising treatment for inflammatory diseases. The immunomodulatory effect of MSCs takes place both by direct cell-to-cell contact and by means of soluble factors that leads to an increased accumulation of regulatory immune cells at the sites of inflammation. Similar efficacy of MSCs has been described regardless of the route of administration used, the inflammation conditions and the major histocompatibility complex context. These observations raise the question of whether the migration of the MSCs to the inflamed tissues is a pre-requisite to achieve their beneficial effect. To address this, we examined the biodistribution and the efficacy of intraperitoneal luciferase-expressing human expanded adipose-derived stem cells (Luci-eASCs) in a mouse model of colitis. Luci-eASC-infused mice were stratified according to their response to the Luci-eASC treatment. According to the stratification criteria, there was a tendency to increase the bioluminescence signal in the intestine at the expense of a decrease in the bioluminescence signal in the liver in the “responder” mice. These data thus suggest that the accumulation of the eASCs to the inflamed tissues is beneficial for achieving an optimal modulation of inflammation.

## 1. Introduction

Mesenchymal stem cells (MSCs) are multipotent adult stem cells that exist in the bone marrow [1], adipose tissue [2], dental pulp [3], and umbilical cord [4], among other tissues [5,6,7]. MSCs have immunomodulatory and regenerative properties allowing their use for treatment of a wide variety of immunological and degenerative disorders [8,9,10,11,12,13,14,15,16]. The immunomodulatory effect of MSCs takes place both by direct cell-to-cell contact and by means of soluble factors [17], although the mechanisms that are involved in their therapeutic effects have not been fully determined. The homing of MSCs has been studied in animal models in a variety of experimental settings (MSC origin, major histocompatibility complex (MHC) context, routes of administration and inflammatory conditions [18,19,20,21,22,23]), the route of administration being the major factor determining the fate of the MSCs in vivo [18,22,24,25,26,27,28,29]. Although a growing number of studies have claimed that MSCs selectively home to sites of injury, regardless of the tissues, the inflammatory conditions and the route of administration [17,18,27,28,30,31], the amount of MSCs that reach the inflammation site is minimal compared to the total number of infused cells. Additionally, similar efficacy of MSCs has been described independently of the route of administration, the inflammation environment and the MHC context. At present, it is unknown whether the therapeutic response to MSC treatments requires the migration of MSCs to sites of inflammation [18,30,32,33] or rather a systemic induction of immune cells with a regulatory phenotype that finally can modulate the ongoing inflammation [9,10,32,34,35]. These observations led us to question whether the accumulation of the MSCs to the site of inflammation is necessary to perform their beneficial effect. To this end, we studied the biodistribution of Luci-eASCs administered intraperitoneally in TNBS-induced colitic mice. Luci-eASC-treated colitic mice were stratified according to their positive response to the MSC treatment.

## 2. Results

### 2.1. Intraperitoneally Administered Luci-eASCs Accumulate Preferentially in the Main Organs and Tissues Independently of the Inflammatory Status of the Mice

To study the biodistribution of the eASCs injected intraperitoneally (IP), luciferase-expressing human expanded adipose derived stem cells (Luci-eASCs) were generated using retrovirus-based methods (Materials and Methods). Phenotype of the Luci-eASCs was confirmed by flow cytometry. Luci-eASCs were positive for CD73, CD90 and CD105 and negative for CD14, CD34, CD45 and HLA-DR according to the criteria of the International Society for Cellular Therapy [18,36] (Supplementary Figure S1A). To evaluate the immunosuppression capacity of Luci-eASCs, in vitro T-cell proliferation assays were carried out using CFSE-labeled PBMCs stimulated with microbeads coated with anti-CD3/CD2/CD28 antibodies in the presence or absence of untransduced eASCs or Luci-eASCs (ratio eASCs:PBMCs, 1:25). Proliferation of viable CD3^+^ T-cells was determined at 120 h by flow cytometry. Luci-eASCs inhibited T-cell proliferation at similar levels as the untransduced eASCs as described in [18] (Supplementary Figure S1B).

**Figure 1:**
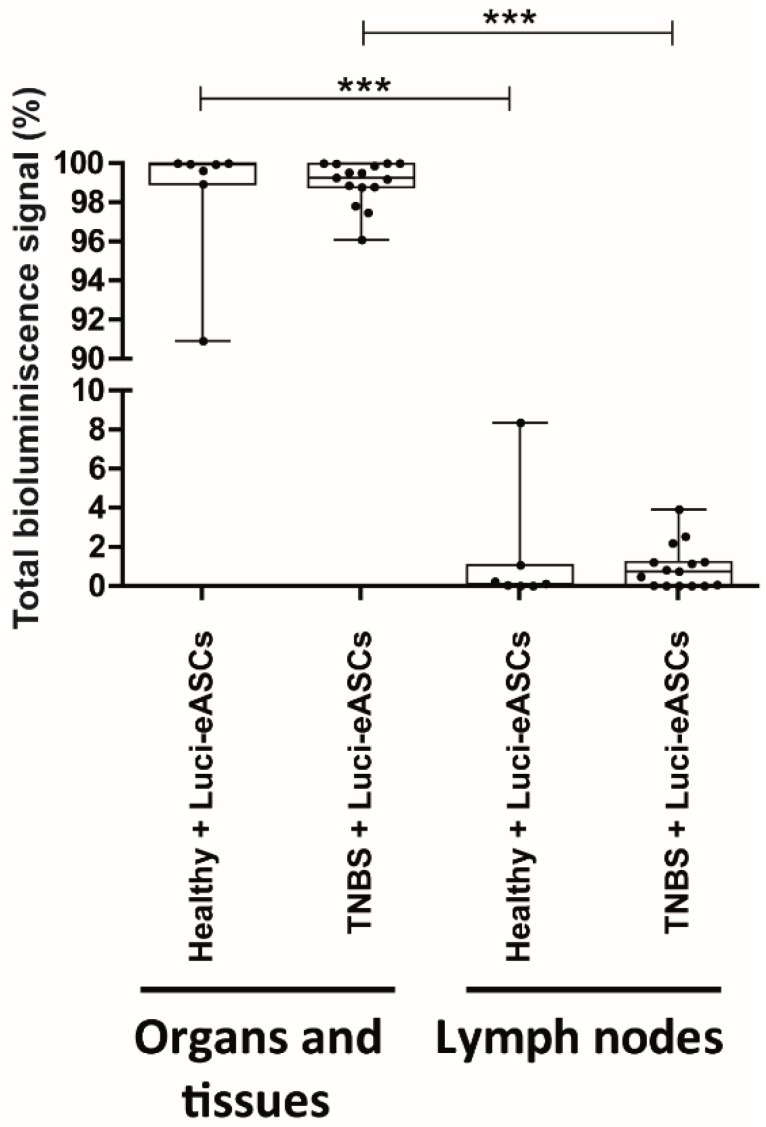
Analysis of total bioluminescence signal in tissues, organs and lymph nodes at 48 h following IP administration of Luci-eASCs in healthy (*n* = 11) and TNBS-colitic mice (*n* = 26). Bioluminescence signals measured at 48 h as percentage of light units in tissues and organs (liver, spleen, intestine, lungs, heart and blood included) and in LNs (inguinal, popliteal, parathymic, parathyroid, mesenteric, caudal and axillary included) relative to the total number of light units per mouse were expressed. Data are presented by dots and box-plots that represent the interquartile range (p75, upper edge; p25, lower edge; p50, midline; p95, line above the box; and p5, line below the box) of the percentage of total bioluminescence signal. Significance was analyzed by the Mann–Whitney *U* test and represented by *** *p* < 0.001. Results correspond to four independent experiments.

**Figure 2:**
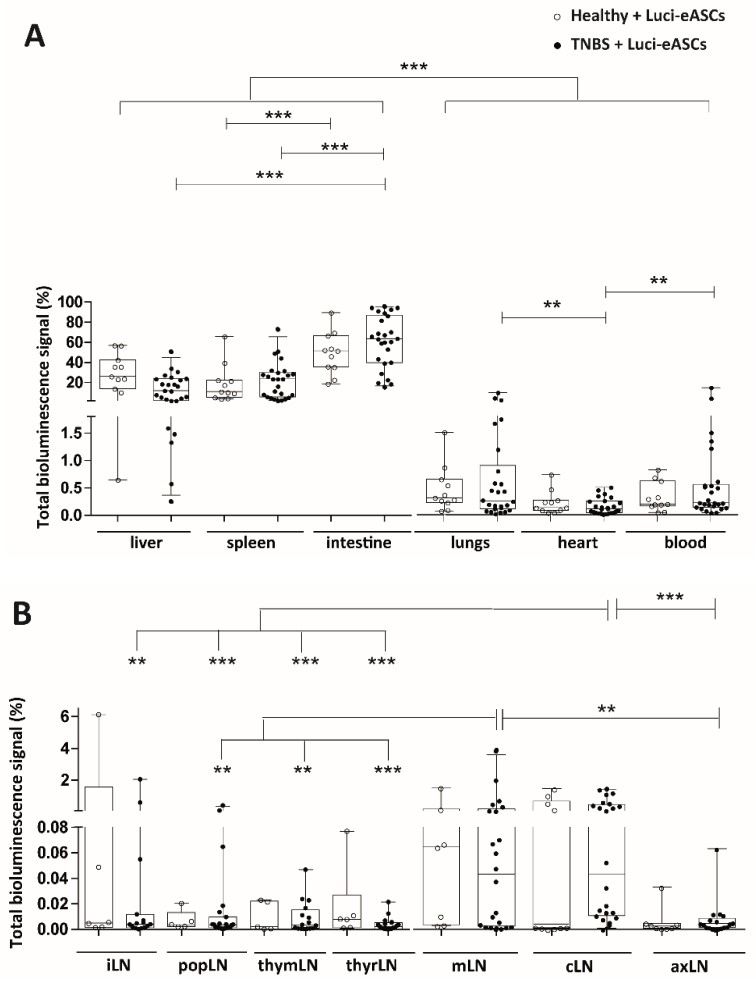
Analysis of bioluminescence signal in different tissues and organs (liver, spleen, intestine, lungs, heart and blood) and different lymph nodes (inguinal, popliteal, parathymic, parathyroid, mesenteric, caudal and axillary) at 48 h following IP administration of Luci-eASCs in healthy and TNBS-colitic mice. Bioluminescence signals measured at 48 h in the liver, spleen, intestine, lungs, heart and blood (**A**) and in the inguinal (iLN), popliteal (popLN), parathymic (thymLN), parathyroid (thyrLN), mesenteric (mLN), caudal (cLN) and axillary (axLN) lymph nodes (LNs). (**B**). Data were expressed as percentage of light units per tissue relative to the total number of light units per mouse. Healthy + Luci-eASCs intraperitoneal (IP), *n* = 11; TNBS + Luci-eASCs IP, *n* = 26. Data are presented by dots and box-plots that represent the interquartile range (p75, upper edge; p25, lower edge; p50, midline; p95, line above the box, and p5, line below the box) of the percentage of total bioluminescence signal. Significance was analyzed by the Mann–Whitney *U* test and represented by ** *p* < 0.007 and *** *p* < 0.001. Results correspond to four independent experiments.

Biodistribution of the Luci-eASCs was assessed in a TNBS-induced colitis mouse model, which displays human inflammatory bowel disease-like clinical, histopathologic and immunologic features [37]. In this model, colitis was induced by intrarectal administration of TNBS and one hour after the inflammatory challenge, Luci-eASCs were administered IP. Biodistribution of Luci-eASCs was measured in the main organs/tissues (liver, spleen, intestine, lungs, heart and blood) and lymph nodes (LNs, inguinal, popliteal, parathymic, parathyroid, mesenteric, caudal and axillary) 48 h after intrarectal administration of TNBS (Figure 1, Figure 2 and Supplementary Figure S2). Initial experiments, following intraperitoneal infusion of Luci-eASCs into healthy mice, were set up to define for how long the bioluminescence signal of the infused Luci-eASCs can be detected in vivo. The bioluminescence signals were monitored at different time points. As shown in Supplementary Figure S2A, the bioluminescence signal declined very rapidly within the first 72 h period from the infusion of the Luci-eASCs. Therefore, 48 h post-infusion of Luci-eASCs was chosen as the best time-point that allows us to analyze simultaneously the in vivo biodistribution and the therapeutic effect of the infused Luci-eASCs.

Most of the bioluminescence signal was detected in the tissues and main organs analyzed (liver, spleen, intestine, lungs, heart and blood) in healthy (99.9[98.9–100]%) and in TNBS-colitic mice (99.2[98.8–100]%, Figure 1 and Supplementary Figure S2C,D). Only a residual amount of bioluminescence was detected in the LNs studied (inguinal, popliteal, parathymic, parathyroid, mesenteric, caudal and axillary) for healthy (0.10[0.01–1.07]%) and TNBS-colitic mice (0.75[0.01–1.74]%).

These results suggest that IP-administered Luci-eASCs have a preference to accumulate to tissues and organs rather than to the LNs, regardless of the inflammatory status of the mice.

### 2.2. TNBS-Colitic Mice Treated with Luci-eASCs Showed an Increase in the Bioluminescence Signal in the Intestine

The bioluminescence signals were also analyzed separately in the different tissues, organs and LNs to further dissect the biodistribution of the IP-administered Luci-eASCs in steady-state and under inflammation.

Luci-eASCs preferentially accumulated in the intestine (51.5[35.4–66.7]% in healthy and 63.5[39.8–87.0]% in TNBS-colitic mice), the liver (26.0[13.7–42.4]% in healthy and 11.7[1.9–23.7]% in TNBS-colitic mice) and the spleen (10.9[5.2–22.2]% in healthy and 23.4[5.6–30.4]% in TNBS-colitic mice). In contrast, very low bioluminescence signals were detected in the lungs, heart and peripheral blood following administration of the Luci-eASCs by IP route (Figure 2A and Supplementary Figure S2C). The increase in the bioluminescence signal found in the intestine in TNBS-colitic mice paralleled by a significant decrease in the bioluminescence signal found in the liver, suggesting that the IP-infused Luci-eASCs under inflammation accumulated preferentially in the intestinal tissue.

As shown in Figure 2B and Supplementary Figure S2D, when Luci-eASCs were administered IP, the bioluminescence signal was nearly undetectable in the LNs analyzed both in healthy and TNBS-colitic mice. There were no clear differences in the bioluminescence signal detected in the LNs analyzed both in healthy and in TNBS-colitic mice. Interestingly, although low, a significant increase in the bioluminescence signal was found in the draining lymph nodes of the colon, 0.043[0.010–0.470]% in cLNs and 0.043[0.002–0.176]% in mLNs, in TNBS-colitic mice with respect to the rest of the LNs analyzed (0.004[0.001–0.012]% for iLNs; 0.003[0.001–0.009]% for popLNs; 0.003[0.001–0.015]% for thymLNs; 0.002[0.001–0.005]% for thyrLNs and 0.004[0.001–0.009]% for axLNs). These results suggest that the preferential accumulation in the mLNs and the cLNs is independent of the inflammatory status of the mice.

Overall, these data indicate that IP infusion of Luci-eASCs favors the accumulation of the Luci-eASCs in the intestine, spleen and liver being practically undetectable within the lymphatic system. When colonic inflammation was induced, an increased amount of bioluminescence signal was found in the intestine in TNBS-colitic mice with a concomitant decrease in the liver.

### 2.3. Luci-eASCs Administered IP Protect Against TNBS-Induced Colitis

**Figure 3:**
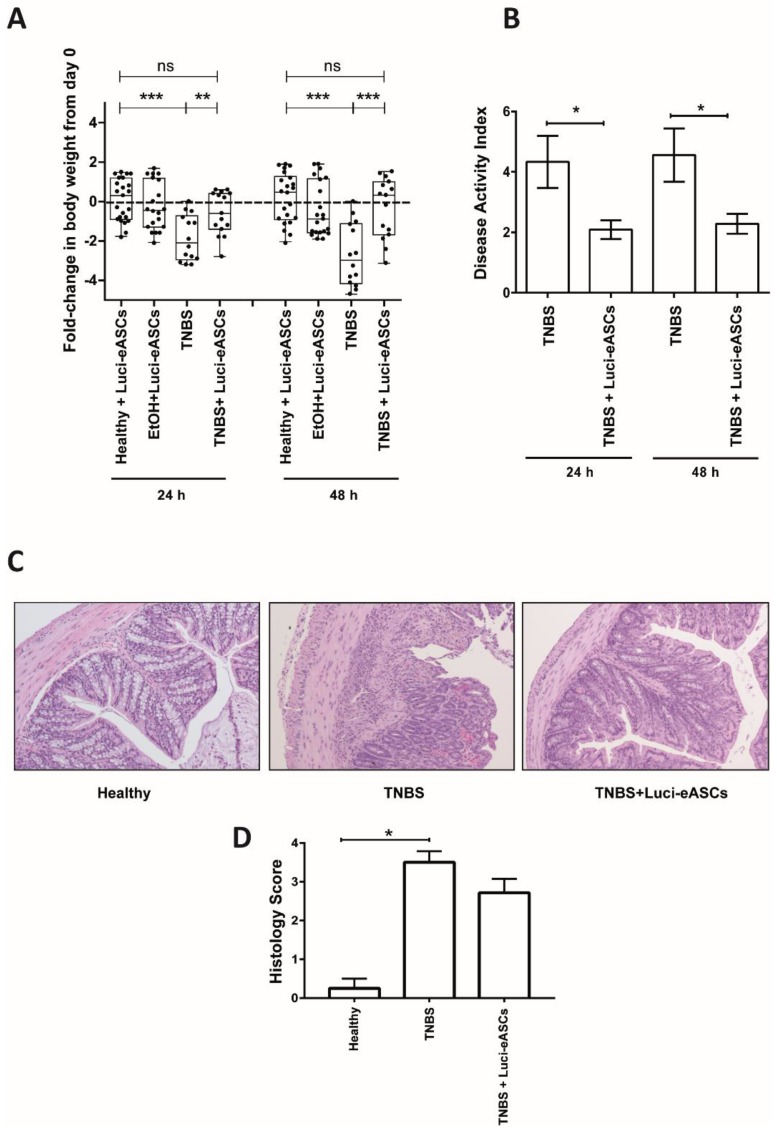
Fold-change in body weights, disease activity index and histological analysis in healthy and TNBS-colitic mice following intraperitoneal (IP) administration of Luci-eASCs. (**A**) Fold-change in body weight at 24 h and 48 h respect to day 0. Healthy + Luci-eASCs, *n* = 22; EtOH + Luci-eASCs, *n* = 20; TNBS, *n* = 14 and TNBS + Luci-eASCs, *n* = 15. Data are presented by dots and box-plots that represent the interquartile range (p75, upper edge; p25, lower edge; p50, midline; p95, line above the box, and p5, line below the box) of the fold-change in body weight at 24 and 48 h with respect to day 0. (**B**) Disease activity index at 24 h and 48 h of TNBS (*n* = 9) and TNBS + Luci-eASCs (*n* = 23). Data are presented by mean and standard error of the mean of disease activity index at 24 h and 48 h. (**C**) Representative images of colon tissue (10× magnification) at 48 h of healthy +Luci-eASCs, TNBS and TNBS + Luci-eASCs mice. (**D**) Histology score of healthy + Luci-eASCs (*n* = 4), TNBS (*n* = 4) and TNBS + Luci-eASCs (*n* = 7). Data are presented by mean and standard error of the mean of histology score at 48 h. Significance was analyzed by the Mann-Whitney *U* test and represented by * *p* < 0.05, ** *p*< 0.01, *** *p* < 0.001 and ns, not significant. Results correspond to four independent experiments.

To know whether the presence of the Luci-eASCs at the site of inflammation can be correlated with their anti-inflammatory and immunomodulatory effect in vivo, the main goal of this study, we analyzed the efficacy of the IP-administered Luci-eASCs in our model of TNBS-induced colitis. To this end, body weights (Figure 3A), disease activity index (Figure 3B) and histological analysis (Figure 3C,D) were conducted following intraperitoneal infusion of Luci-eASCs.

As expected, TNBS-treated mice had a significant reduction in the body weights at 24 h (−2.1[−2.9 to −0.72] fold-change in body weight) and 48 h (−3.0[−4.2 to −1.1] fold-change in body weight) with respect to the control groups (healthy and mice treated with intrarectal 50% EtOH, vehicle of the TNBS) (Figure 3A). Importantly, colitic mice treated with Luci-eASCs had a decrease in their body weights significantly less pronounced than the untreated colitic group at 24 h (−0.6[−1.4 to 0.4] fold-change in body weights) and, more significantly, at 48 h (0.3[−1.7 to 1.0] fold-change in body weights), when the mice started to regain weight (Figure 3A).

Similarly, all Luci-eASC-treated colitic mice had a significant reduction in the disease activity index at 24 and 48 h (2.1 ± 0.3 and 2.3 ± 0.3, respectively) with respect to the TNBS-colitic mice (4.3 ± 0.9 and 4.6 ± 0.9, respectively, Figure 3B). Additionally, the severity of colitis was evaluated in colon tissue by hematoxylin and eosin staining. Colitic mice exhibited significant alterations in colon structure with massive infiltration of mononuclear cells. In contrast, the treatment with Luci-eASCs preserved the colon morphology, reduced the extent of the inflammatory areas and attenuated the leukocyte infiltration (Figure 3C,D).

Overall, these results suggest that a single dose of Luci-eASCs injected IP can modulate the acute inflammatory responses at both 24 h and 48 h in an experimental model of the TNBS-induced colitis.

### 2.4. Comparative Analysis in the Biodistribution and the Therapeutic Effects of the Luci-eASCs

**Figure 4:**
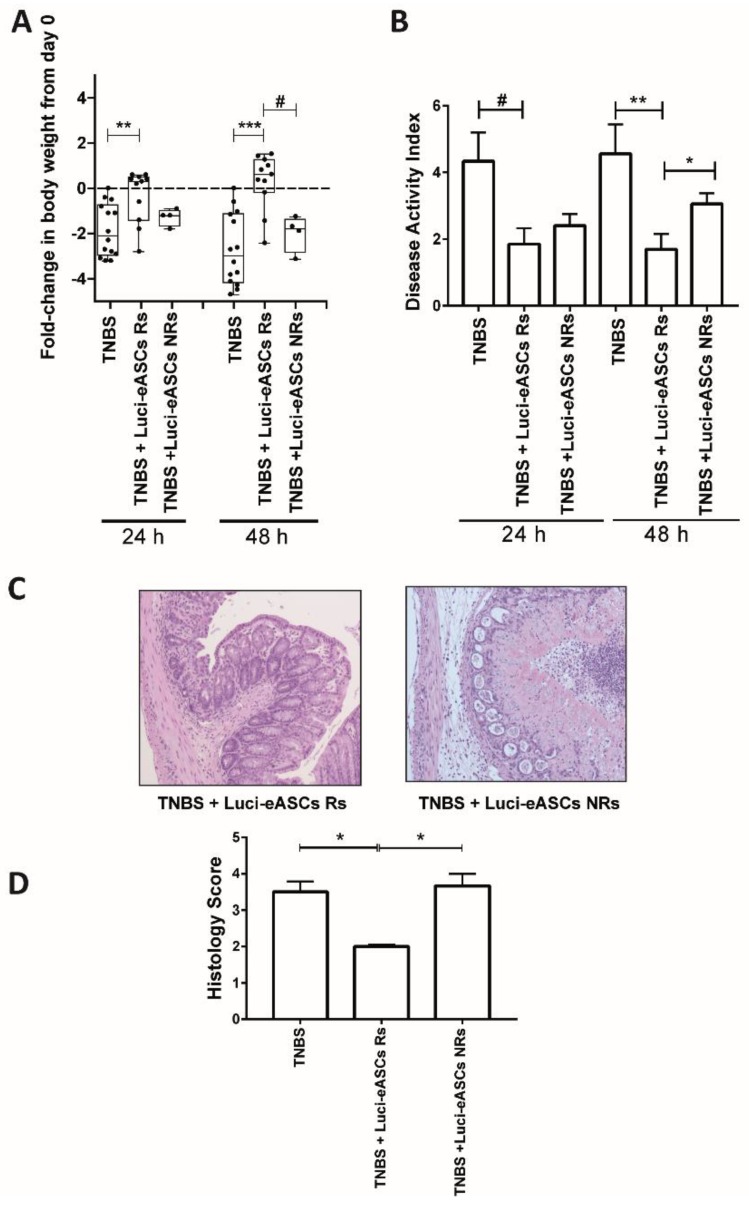
Stratification of in vivo responses to treatment with Luci-eASCs administered IP in TNBS-colitic mice according to the fold-change in body weights and disease activity index, from 24 h to 48 h, and histological analysis at 48 h. Colitic mice treated with Luci-eASCs were grouped into “responders” (Rs mice that did not lose weight at 48 h with respect to 24 h) and “non-responders” (NRs mice that lost weight at 48 h with respect to 24 h). (**A**) Fold-change in body weights at 24 h and at 48 h. TNBS, *n* = 14; TNBS + Luci-eASCs Rs, *n* = 9 and TNBS + Luci-eASCs NRs, *n* = 6. Data are presented by dots and box-plots that represent the interquartile range (p75, upper edge; p25, lower edge; p50, midline; p95, line above the box, and p5, line below the box) in fold-change in body weights. (**B**) Disease activity index at 24 h and 48 h of TNBS (*n* = 9), TNBS + Luci-eASCs Rs (*n* = 13) and TNBS + Luci-eASCs NRs (*n* = 10) mice. Data are presented by mean and standard error of the mean of disease activity index at 24 h and 48 h. (**C**) Representative images of colon tissue (10×) at 48 h of TNBS, TNBS + Luci-eASCs Rs and TNBS + Luci-eASCs NRs. (**D**) Histology score of TNBS (*n* = 14), TNBS + Luci-eASCs Rs (*n* = 4) and TNBS + Luci-eASCs NRs (*n* = 3). Data are presented by mean and standard error of the mean of histology score at 48 h. Significance was analyzed by the Mann-Whitney U test and represented by # *p* ≤ 0.025, * *p* < 0.05, ** *p* < 0.01 and *** *p* < 0.001. Results correspond to four independent experiments.

To dissect whether the presence of the Luci-eASCs at the inflammation site can be correlated directly to their modulatory effect in vivo, the fold-change in body weight between 24 h and 48 h were used as a parameter to stratify mouse responses to treatment with Luci-eASCs. In this sense, “responder” TNBS colitic-mice were those mice that did not lose weight between 24 h and 48 h post-infusion of the Luci-eASCs. “Non-responder” TNBS colitic-mice were those mice that lost weight at 48 h after the infusion of the Luci-eASCs with respect to 24 h (Figure 4). According to these criteria, 9 out of the 15 mice had a positive response to the treatment which represents 60% positive response to the treatment with Luci-eASCs (Figure 4A). As shown in Figure 4A, the “responder” mice gained weight, at both 24 and 48 h with respect to the untreated colitic group of mice and the “non-responder” mice. In accordance to these results, the “responder” mice had a significant reduction of the disease activity index at 24 and 48 h (1.8 ± 0.5 and 1.7 ± 0.5, respectively) with respect to the TNBS-colitic mice (4.3 ± 0.9 and 4.6 ± 0.9, respectively) as well as to the “non-responder” mice (3 ± 0.3) at 48 h (Figure 4B). In accordance to this, the histological analysis of the colon showed that the “responder” mice had a significantly attenuated loss of colon structure and cell infiltration with respect to the “non-responder” mice (Figure 4C,D).

**Figure 5:**
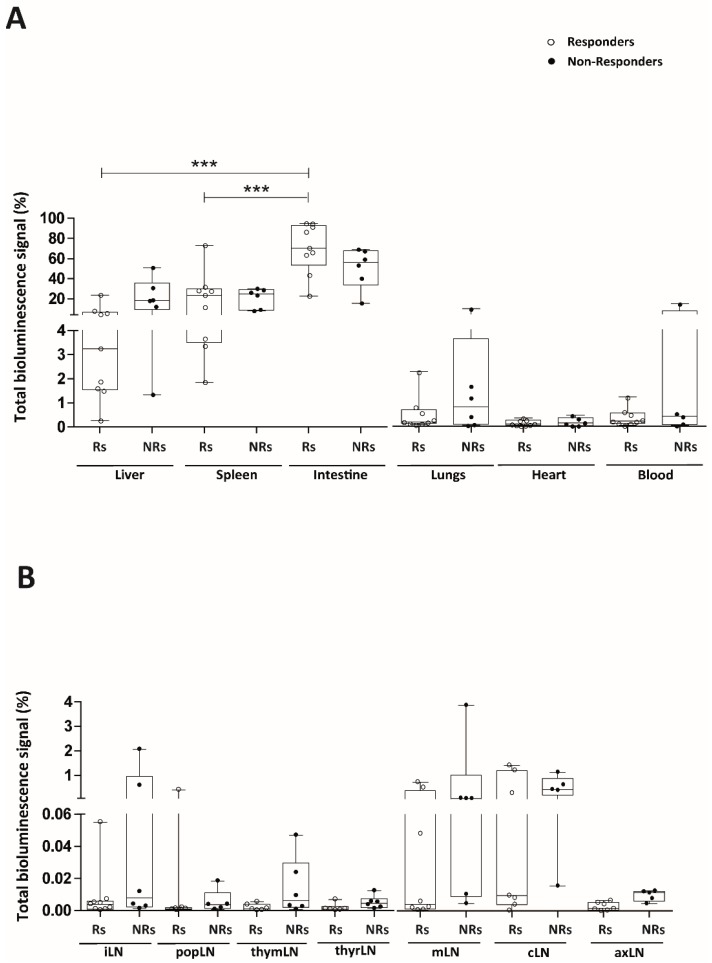
Analysis of the bioluminescence signal in the different tissues and organs (liver, spleen, intestine, lungs, heart and blood) and in the different lymph nodes (inguinal, popliteal, parathymic, parathyroid, mesenteric, caudal and axillary) at 48 h in “responder” and “non-responder” TNBS-colitic mice. (**A**) Bioluminescence signals, measured at 48 h, in the liver, spleen, intestine, lungs, heart and blood and in the inguinal (iLN), popliteal (popLN), parathymic (thymLN), parathyroid (thyrLN), mesenteric (mLN), caudal (cLN) and axillary (axLN) LNs. (**B**) in “responders” (did not lose weight at 48 h with respect to 24 h) and “non-responders” (lost weight at 48 h with respect to 24 h) were analyzed as percentage of light units per tissue relative to the total number of light units per mouse. TNBS + Luci-eASCs Rs, (*n* = 9); TNBS + Luci-eASCs NRs, (*n* = 6). Data are presented by dots and box-plots that represent the interquartile range (p75, upper edge; p25, lower edge; p50, midline; p95, line above the box, and p5, line below the box) of the percentage of total bioluminescence signal. Significance was analyzed by the Mann-Whitney *U* test represented by *** *p* < 0.001. Results correspond to four independent experiments.

To define whether a correlation exists between the immunomodulation of the intestinal inflammation and the in vivo biodistribution of the Luci-eASCs, we compared the bioluminescence signals in the tissues, organs and in the LNs in the “responder” and “non-responder” mice separately (Supplementary Figure S3 and Figure 5). In this sense, “responder” mice tended to have an increase in the bioluminescence signal in the tissues and organs analyzed (99.5[99.0–100]%) with respect to the “non-responder” mice (98.8[97.1–99.4]%, Supplementary Figure S3), although the differences observed were not significant. This paralleled to a tendency to decrease the bioluminescence signal in the LNs in the “responder” mice (0.035[0.009–0.734]%) compared to the bioluminescence signal in the LNs of the “non-responder” mice (1.189[0.568–2.878]%) suggesting that the Luci-eASCs tend to remain in the organs and tissues instead of accumulating within the lymphatic system to perform their beneficial effect.

The “responder” mice have a significant accumulation of the total bioluminescence signal in the intestine (70.3[53.4–92.9]%) with respect to the spleen (23.4[3.5–29.8]%) and the liver (3.2[1.5–6.6]%). Moreover, the bioluminescence signal of the intestine in the “responder” mice tended to increase with respect to the “non-responder” mice (56.2[33.9–67.7]%), although this difference did not reach statistical significance. This was in parallel to a tendency to decrease the total bioluminescence signal in the liver in the “responder” (3.2[1.5–6.6]%) with respect to the “non-responder” mice (18.2[9.3–35.8]%), again not significantly. No differences were found in the spleens in “responder” mice (23.4[3.5–29.8]%) with respect to the spleens in the “non-responder” mice (24.8[8.8–29.0]%) (Figure 5A).

When the bioluminescence signal was analyzed separately in the different LNs in the “responder” and “non-responder” mice, although low, a tendency to decrease the bioluminescence signal in the different lymph nodes analyzed in the “responder” with respect to the “non-responder” mice was observed (Figure 5B).

Altogether, these results suggest that the slight increase accumulation of the eASCs at the inflamed colonic tissue in “responder” mice may explain, at least in part, their positive response to the cell therapy treatment with the eASCs.

## 3. Discussion

The biodistribution of MSCs has been studied in a variety of animal models and experimental settings (tissue origin of MSC, MHC context, inflammatory conditions and route of administration [18,20,21,25,28,33]). Although several studies claimed that, under inflammatory conditions, MSCs selectively home to sites of injury [17,27,30,31], this preferential homing to the inflamed tissue is controversial, since the biodistribution of the MSCs is strongly dependent on the route of administration used and, in general, the proportion of MSCs that reach the inflamed tissue, respect to the total amount of administered MSCs, is minimal. This may imply that the presence of MSCs at the site of inflammation is not necessarily required to perform their beneficial effect. On the other hand, the efficacy of the treatment with MSCs does not seem to be altered by the route of administration and the inflammatory status of the mice [25,38]. To confirm whether the presence of MSCs at the site of inflammation is necessary to achieve a beneficial effect, we analyzed the biodistribution and the efficacy of IP-administered human eASCs in a TNBS-induced colitis stratifying the mice according to their positive response to the eASC treatment. This analysis could provide valuable information when studying the correlation between the presence at the site of inflammation and the efficacy of the MSCs over indiscriminate analysis of biodistribution of MSCs in an entire experimental population [20,28,33]. The analysis was done 48 h post-infusion of Luci-eASCs as the optimal time-point that allows us to analyze simultaneously the in vivo biodistribution and the therapeutic effect of the infused Luci-eASCs. According to the stratification, based on the positive response to the eASC treatment, a slight correlation between the presence of the MSCs in the intestine and the immunomodulatory effects of eASCs was observed, although these differences were not statistically significant.

The comparative analysis of the biodistribution of IP administered Luci-eASCs, in healthy and TNBS-colitic mice allow us to analyze the homing of the MSCs to the inflamed tissue. In agreement with previous reports [20,28,33,39,40], the majority of the Luci-eASCs accumulated preferentially in the intestine, the spleen and the liver when the Luci-eASCs were infused IP (Figure 1 and Figure 2). A reduced amount of bioluminescence signal was detected in the lungs, heart, peripheral blood and LNs (Figure 1 and Figure 2). The presence of the Luci-eASCs increased significantly in the intestine that paralleled a decrease in the liver in the TNBS-colitic mice, suggesting the preferential accumulation of MSCs to the site of injury. These results are in accordance with Gonzalez MA et al. [33] and Castelo-Branco MTL et al. [28] that claimed the homing of the IP-administered MSCs to the inflamed colon with respect to the non-inflamed colon, in a similar model of TNBS-induced colitis. Interestingly, Gonzalez MA and Castelo-Branco MTL used, not only xenogeneic, but also allogeneic and syngeneic MSCs. Their results suggest that the accumulation of the MSCs at the site of inflammation is independent of the MHC context. In this sense, it has been demonstrated that syngeneic and allogeneic mouse MSCs show higher survival rates than xenogeneic MSCs with no clear differences in their efficacy and homing [22,41,42,43].

Within the marginal amount of the bioluminescence signal found in the LNs analyzed, the IP-infused Luci-eASCs preferentially accumulate in mLNs and cLNs independently of the inflammatory status of the mice. To the best of our knowledge this is the first study where the biodistribution of the IP-administered MSCs within the lymphatic system has been analyzed systematically. Gonzalez MA et al. [33] and Anderson P et al. [44], using a similar TNBS-induced colitis mouse model, and Wang M et al. [20], in a DSS colitic mouse model, found the presence of MSCs in the mLNs in colitic mice following IP administration. Other reports have also demonstrated the presence of MSCs in the mLNs in a multiorgan autoimmune model where MSCs were infused IP43. In all instances, the proportion of MSCs found in the mLNs was rather low with respect to the total MSCs dose infused into recipient mice. All these studies demonstrated that, regardless of the inflammatory status and the route of administration, a marginal amount of MSCs can be found within the lymphatic system. In our study, we analyzed different LNs in healthy and in colitic mice showing that, within the lymphatic system, the Luci-eASCs preferentially accumulate in the mLNs and cLNs after IP administration, suggesting that the preferential accumulation of the MSCs to the draining LNs is mainly dependent on the route of administration and less affected by the inflammatory environment. Additionally, in a recent study, following intralymphatic (IL) administration of Luci-eASCs, we have demonstrated that although the majority of the cells remained at the site of injection a small but significant bioluminescence signal was increased in the intestine in colitic mice with respect to the healthy mice [18], further supporting the notion that MSCs tend to accumulate at the inflammation site [20,28] regardless of the route of administration used.

To answer the question about the need for the presence of the infused MSCs to the site of inflammation to achieve a beneficial effect, we studied the correlation between the biodistribution of the Luci-eASCs and their efficacy in modulating the acute inflammatory challenge. To this end, Luci-eASC-treated colitic mice were stratified according to their response to the cell therapy treatment. It should be noticed that the biodistribution analysis was conducted using a single dose of the Luci-eASCs whereas the majority of studies aiming at modulating immune responses used multiple eASCs infusions in order to achieve a very robust modulation of inflammation [33,45]. According to the stratification criteria, we found that there was a tendency to increase the bioluminescence signal in the intestine at the expense of a decrease in the bioluminescence signal in the liver (Figure 5 and Supplementary Figure S3) in the “responder” mice. This result was accompanied by a reduction in the bioluminescence signal in the LNs, suggesting that the presence of Luci-eASCs in the site of inflammation is required to perform their therapeutic effects.

In contrast to these results, we have previously demonstrated that, following intranodal administration of MSCs, we could not establish any correlation between the presence of the MSCs in the intestine and the modulation of the inflammation; although, similar degree of positive response to the treatment with Luci-eASCs was achieved. These observations further confirm that the beneficial effect of the MSCs is independent of the route of administration used [18].

In line with these observations, it should also be highlighted that there are controversial data about the optimal route of administration for MSCs and their efficacy in modulating experimental colitis. Similar efficacy of MSCs using intravenous (IV), IP and IL administration has been demonstrated under different inflammatory conditions [18,25,34,38], suggesting that the biodistribution of the MSCs is strongly dependent on the route of administration. Goncalve FC et al. demonstrated that the IV administration of MSCs is a superior method for reducing colon inflammation compared with intraperitoneal therapy [38]. In contrast to these results, Wang M et al. [20] and Castelo-Branco MTL et al. [28] demonstrated that the IP administration of MSCs was more efficacious than intra-anal and IV administration of MSCs in ameliorating colitis. Their conclusions were based on the quantity of MSCs that reaches the inflamed colon and, in this sense, it has been established that local administration of MSCs, such as intra-anal infusion of MSCs, is an effective treatment for experimental colitis [20]. Our results, along with findings in previous studies showed similar efficacy of MSCs regardless of the route of administration, the inflammation conditions and the MHC context. It has been previously described that the reduction of systemic inflammation in this experimental model of colitis is mediated by the induction of regulatory CD25^+^Foxp3^+^CD4^+^ T cells in the colon and mLNs [33,46] together with a reduction in the levels of IL17 in the lamina propria mononuclear cells [45]. Furthermore, we have recently found that endoscopic administration of eASCs in the colon submucosa of TNBS-colitic rats attenuated the severity of colitis and reduced inflammation, which was associated to increased expression of Foxp3 and IL−10 mRNA in mLNs of eASC-treated rats [47].

## 4. Materials and Methods

### 4.1. Mice

C57/BL6 male mice (6–8 weeks) were obtained from Charles River. All experiments were performed in accordance with the corresponding regulations regarding experimental animal welfare (RD 223/1998 and Directive 2010/63/EU protocols). The experimental protocol was reviewed and approved by the ethics committee for animal research of the CIEMAT and Comunidad de Madrid (based on the RD 53/2013).

### 4.2. Generation of Human Expanded Adipose-Derived Stem Cells

Human samples were obtained after informed consent as approved by the Spanish Ethics Committee of reference for the site of tissue procurement (Clínica de la Luz Hospital, Madrid, Spain). Human adipose tissue aspirates from healthy donors were processed as described elsewhere [18]. Briefly, human adipose tissue aspirates from healthy donors were washed with phosphate-buffered saline and digested with 0.075% collagenase (Type I, Invitrogen, Carlsbad, CA, USA). The digested sample was washed with fetal bovine serum (FBS) and NH_4_Cl to eliminate remaining erythrocytes and suspended in culture medium (Dulbecco’s modified Eagle medium with 10% FBS). Cells were seeded in tissue culture flasks and expanded (37 °C, 5% CO_2_). Cells were transferred to a new flask when they reached 90% confluence. Experiments were performed with a pool of cells from three male and three female adult donors at population doublings 12–14. All the eASCs used fulfilled the release criteria of identity, purity and potency needed for their clinical use.

### 4.3. Generation of Luciferase^+^ Transduced eASCs (Luci-eASCs)

Reporter Luciferase-EGFP bicistronic retroviral vector was constructed using standard cloning procedures as described [18]. Briefly, the H2B gene was amplified by PCR (forward, TATGGGTACCGCCACCATGCCAGAGCCAGCGAAG; reverse, TATGGATCCTTAGCGCTGGTGTACTTG) and cloned into pCopGFP2i-N (Evrogen, Moscow, Russia) using Acc65I–BamHI sites. Internal Ribosome Entry Site (IRES) element was isolated from pIRES2-EGFP (Clontech, Mountain View, CA, USA) using SalI–MscI and cloned upstream H2B-CopGFP2i into SalI–Acc65I (Klenow blunted) sites. Luciferase was cut from pGL2 Basic (Promega, Madison, WI, USA), using XhoI–XbaI (Klenow blunted) restriction sites, and inserted upstream IRES-H2B-CopGFP2i into XhoI–SalI (Klenow blunted). CopGFP2i was then replaced by EGFP, from pEGFP-N1 (Clontech) using AgeI–NotI sites. The Luciferase-IRES-H2B-EGFP cassette was separated using Bgl II–NotI and cloned into BamHI–NotI sites of pRV retroviral vector to give the final pRV-Luciferase-IRES-H2B-EGFP bicistronic reporter construction. Transfection and generation of viral supernatants were performed using polyethylenimine (1vol PEI: 2 vol DNA). Viral supernatants were used for infection of eASCs. The eASCs were infected after seeding the eASCs in 6-well plates and infected with retroviral particles concentrated when cell confluency was in the range of 70%–80%. Finally, the transduction was evaluated by flow cytometry (FACS Calibur™, BD Bioscience, San Diego, CA, USA), selecting clones with 90% expression of EGFP by cell sorting.

### 4.4. Immunophenotyping of Luci-eASCs

Luci-eASCs were defined according to the criteria of the International Society for Cellular Therapy [36]. Luci-eASCs were stained using antibodies for CD73 (AD2), CD90 (5E10, both from Becton Dickinson), CD105 (43A3, Biolegend, San Diego, CA, USA), CD14 (RM052, Immunotech, Monrovia, CA, USA), CD34 (8G12, Becton Dickinson, Franklin Lakes, NJ, USA), CD45 (J33, Beckman Coulter, Brea, CA, USA) and HLA-DR (AF6-120.1, eBiosciences, Waltham, MA, USA) [18].

### 4.5. Immunosuppression Assay of Luci-eASCs

Buffy coats were provided by the National Transfusion Centre of the Comunidad Autonoma of Madrid, Spain. Peripheral blood mononuclear cells (PBMCs) were isolated by density centrifugation gradient using Ficoll-Paque Plus (GE Healthcare Biosciences AB, Uppsala, Sweden) following the supplier’s protocol.

PBMCs (2 × 10^7^) were resuspended in 10 mM of 5(6)-carboxyfluorescein diacetate N-succinimidyl ester (CFSE, Molecular Probes, Eugene, OR, USA) solution and incubated at 37 °C for 10 min. Reaction was stopped by adding ice-cold medium (RPMI + 10% FBS) and cells were washed with ice-cold phosphate buffer saline. After resting overnight, CFSE-labeled PBMCs were cultured in 24-well plates alone or with Luci-eASCs (4 × 10^4^ cells/well; Luci-eASC:PBMC ratio 1:25) in a RPMI+10% FBS and were activated with the Pan T Cell Activation Kit (microbeads coated with anti-CD3, anti-CD2 and anti-CD28; Miltenyi Biotech, Bergisch Gladbach, Germany) following the manufacturer’s instructions [48,49]. After 5 days, PBMCs were harvested, labeled with 7-AAD and anti-CD3 antibody and cell proliferation of the CD3^+^/7-AAD^−^ population (viable CD3 T lymphocytes) was determined by flow cytometry, according to the decrease in the CFSE fluorescence intensity. Data were analyzed with the use of FCSExpress 4 software (De Novo Software, Los Angeles, CA, USA) [18].

### 4.6. Induction and Evaluation of Colitis after Treatment with Luci-eASCs

To induce colitis, anaesthetized mice were preimmunized on their shaped back with 1% of trinitrobenzene sulfonic acid (TNBS, Sigma-Aldrich, Saint Louis, MI, USA). After one week, colitis was induced by intrarectal administration of 3 mg of TNBS in 50% ethanol (100 µL) per mouse. One hour after, TNBS administration, 3.2 × 10^5^ of Luci-eASCs per mouse were administered IP. As controls, healthy mice with the Luci-eASC treatment and preimmunized mice with intrarectal 50% EtOH (vehicle of the TNBS) with the IP Luci-eASC treatment. Colitis score (weight of mice, stools and general aspect of mice) was monitored for 48 h. The fold-change in body weights were calculated by the difference with respect to the initial body weight at day 0. Disease activity score was defined as follows: (1) Body weight loss (0: no loss; 1: 1%–5%; 2: 5%–10%; 3: 10%–20% and 4: >20% loss of weight); (2) stool consistency (0: normal stools; 2: loose stools; 3: watery diarrhea and 4: watery diarrhea with blood) and (3) the general activity (0: normal; 1–2: moderate activity; 3: null activity and 4: no survival).

### 4.7. Histological Analysis

Colons were surgically removed and fixed with formalin overnight at 48 h, following the TNBS administration. 1-cm colon tissues cut were collected. Colon sections were embedded in paraffin and stained with hematoxylin/eosin. The sections were microscopically examined for histopathological changes using the following scoring system (0: no evidence of inflammation; 1–2: low level of inflammation with scattered infiltrating mononuclear cells; 3: high level of inflammation with mild loss of structure 4: maximal severity of inflammation with massive leukocyte infiltration and total loss of structure).

### 4.8. In Vivo Optical Bioluminescence Imaging

Bioluminescence imaging analysis was conducted at 48 h, following the infusion of the Luci-eASCs, with the IVIS 200 imaging system (Caliper). Whole body bioimaging analysis was done in anesthetized mice. The bioimaging analysis in main organs, tissues (liver, spleen, intestine, lungs, heart and blood) and secondary LNs (inguinal, iLN, popliteal, popLN; parathymic, thymLN; parathyroid, thyrLN; mesenteric, mLN; caudal, cLN and axillary, axLN, LNs) were determined immediately after culling the mice. Photons emitted were acquired as photons per s/cm^2^ per steradian using Living Imaging 3.0 (Caliper). For photon quantification, a region of interest was manually selected and kept constant within each experiment.

Bioluminescence signal was analyzed as percentage of light units per tissue relative to the total number of light units per mouse. Total bioluminescence signal for tissues, organs and LNs was calculated as the sum of the light units in each tissue. Values of bioluminescence signal below 10,000 light units were considered negative.

### 4.9. Statistical Analysis

Data are presented as the interquartile range, (p75, upper edge; p25, lower edge; p50, midline; p95, line above the box, and p5, line below the box) and the mean and standard error of the mean (SEM). Normal distribution was analyzed by the Shapiro-Wilks test. Non-parametric techniques (Mann-Whitney U test) were used (with Bonferroni adjustment). Analysis was performed using the software Stata 11 (StataCorp. College Station, TX, USA) and GraphPad Prism 7.00 (GraphPad Software, La Jolla, CA, USA).

## 5. Conclusions

Our results indicate that when eASCs are administered IP in colitic mice, the IP-infused MSCs tend to accumulate in the inflamed intestine to perform their therapeutic effect. The stratification analysis based on the response to MSC treatment provides a very useful approach that may help to clarify the mechanisms mediating the therapeutic effects of MSCs and, hence, improve the use of MSC-mediated therapies in the clinic.

## Supplementary Materials

The following are available online at http://www.mdpi.com/1422-0067/19/7/1853/s1.
