# Supplementary material for: Comparative Analysis between the In Vivo Biodistribution and Therapeutic Efficacy of Adipose-Derived Mesenchymal Stromal Cells Administered Intraperitoneally in Experimental Colitis
Source: Int J Mol Sci. 2018 Jun 23;19(7):1853. doi: 10.3390/ijms19071853 (PMC6073850; doi:10.3390/ijms19071853)
Supplement: Supplementary file 1 [file ijms-19-01853-s001.pdf]

Supplementary Materials

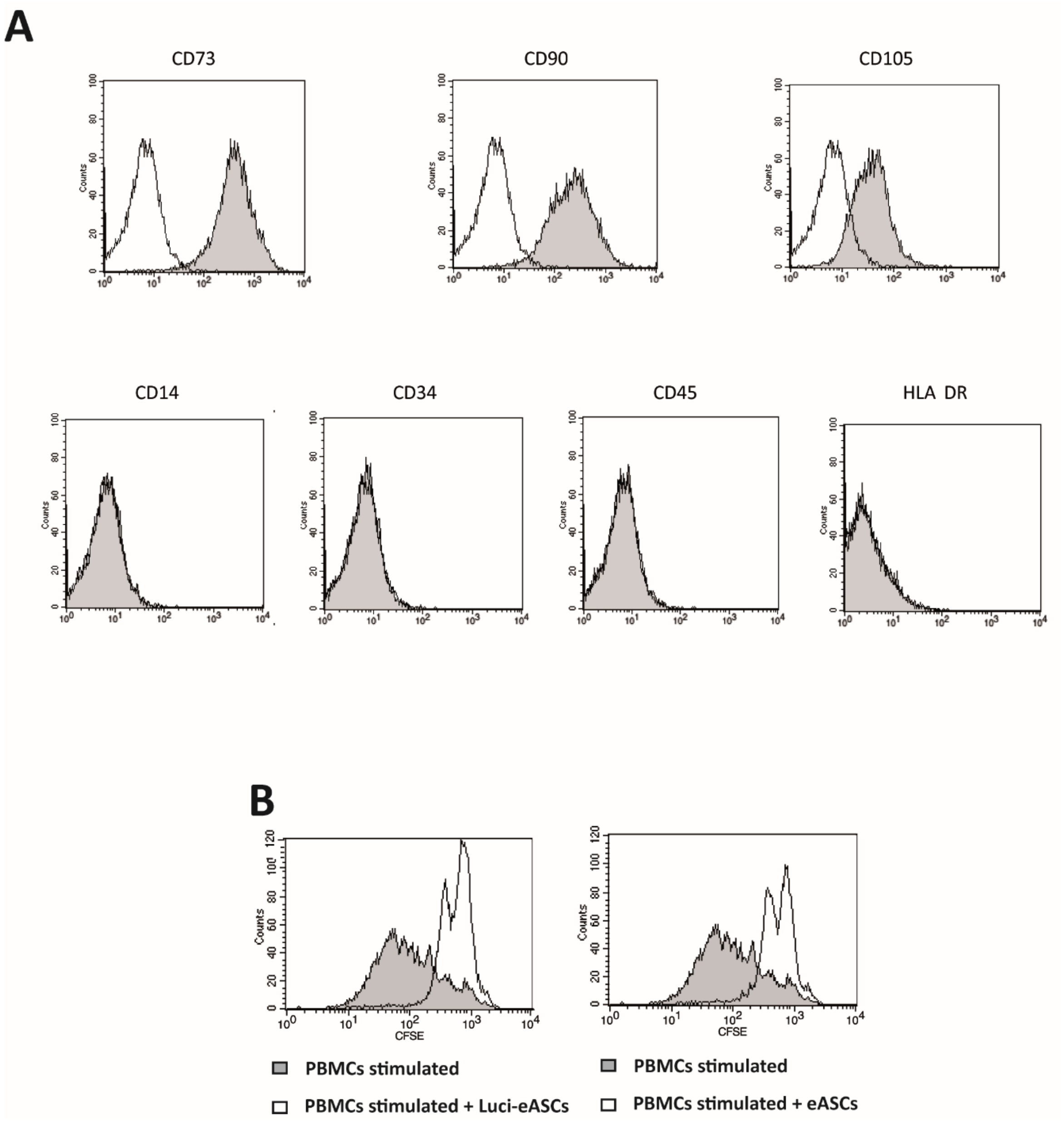

Figure S1. Characterization of Luci-eASCs. (**A**) Luci-eASCs were analyzed by flow cytometry using antibodies against CD73, CD90, CD105, CD14, CD34, CD45 and HLA-II, (grey color). The corresponding isotype controls for each surface marker are shown (white color). Histograms shown are representative of three experiments. (**B**) Representative histograms of T-lymphocyte proliferation are shown. CFSE-labeled PBMCs were stimulated with anti-CD3/CD2/CD28 coated beads in the absence (grey color) or presence (white color) of transduced eASCs (Luci-eASCs, left) or untransduced eASCs (right), ratio 1:25 eASCs or Luci-eASC:PBMCs. Proliferation of the viable $CD3^{+}$ T cells was monitored after 120 h by flow cytometry.

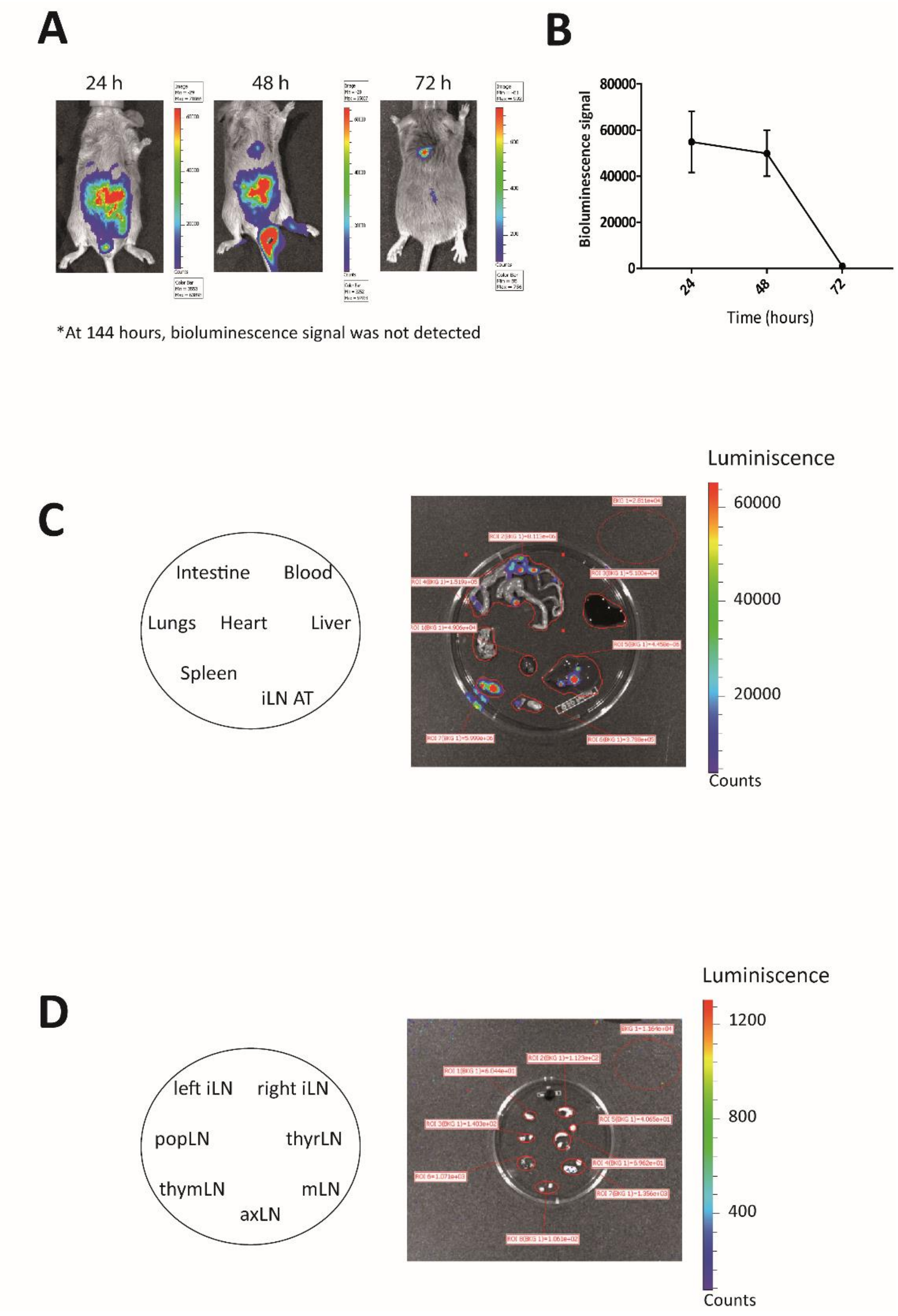

Figure S2. In vivo imaging of IP administered Luci-eASCs in healthy mice. (**A**) Representative images of total bioluminescence signal at 24, 48 and 72 hours when Luci-eASCs were administered intraperitoneally in healthy mice. (**B**) Graph represents the decline of total bioluminescence signal (luciferase activity) at 24h, 48h and 72 h. (**C**) Representative image of bioluminescence signal at 48 hours in intestine, blood, lungs, heart, liver, spleen and adipose tissue adjacent inguinal lymph nodes (iLN AT). (**D**) Representative image of the bioluminescence signal at 48 hours in left and right inguinal (iLN), popliteal (popLN), parathyroid (thyrLN), parathymic (thymLN), mesenteric (mLN) and axillary (axLN) lymph nodes.

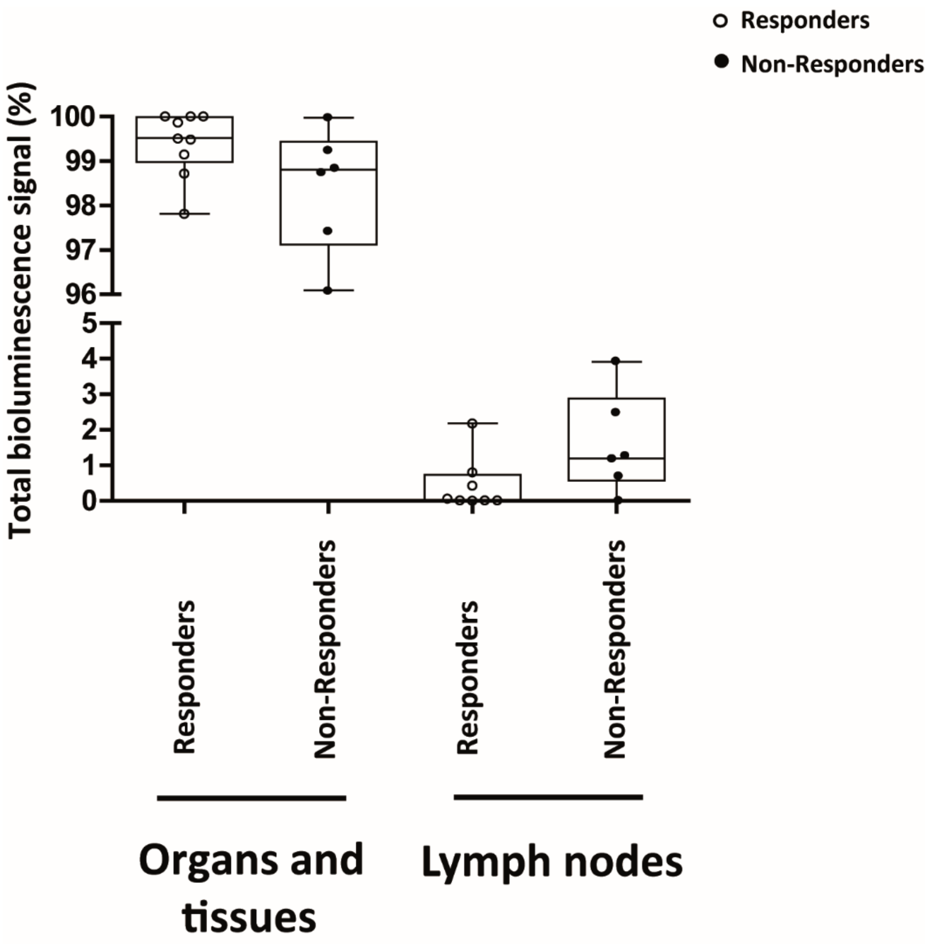

Figure S3. Analysis of the bioluminescence signal in tissues, organs and lymph nodes, at 48 hours in "responder" (R) and `non-responder´ (NR) TNBS-colitic mice treated with IP-administered Luci-eASCs. Bioluminescence signals measured at 48 h as percentage of light units per each tissue and organ (liver, spleen, intestine, lungs, heart and blood included) and per LN (inguinal, popliteal, parathymic, parathyroid, mesenteric, caudal and axillary included) relative to the total number of light units per mouse were analyzed. TNBS + Luci-eASCs IP Rs (n = 9); TNBS + Luci-eASCs IP NRs (n = 6). Data are presented by dots and box-plots that represent the interquartile range (p75, upper edge; p25, lower edge; p50, midline; p95, line above the box, and p5, line below the box) of the percentage of total bioluminescence signal. Significance was analyzed by the Mann-Whitney U test. Results correspond to four independent experiments.
